# Neuroanatomical Alterations in Patients With Tinnitus Before and After Sound Therapy: A Voxel-Based Morphometry Study

**DOI:** 10.3389/fnins.2020.00911

**Published:** 2020-09-08

**Authors:** Xuan Wei, Han Lv, Zhaodi Wang, Chunli Liu, Pengling Ren, Peng Zhang, Qian Chen, Yawen Liu, Pengfei Zhao, Shusheng Gong, Zhenghan Yang, Zhenchang Wang

**Affiliations:** ^1^Department of Radiology, Beijing Friendship Hospital, Capital Medical University, Beijing, China; ^2^Department of Otolaryngology Head and Neck Surgery, Beijing Friendship Hospital, Capital Medical University, Beijing, China

**Keywords:** tinnitus, sound therapy, thalamus, voxel-based morphometry, functional magnetic resonance imaging

## Abstract

According to previous studies, many neuroanatomical alterations have been detected in patients with tinnitus. However, few studies have reported on the morphological changes observed following sound therapy. To explore the brain anatomical alterations in patients with idiopathic tinnitus using voxel-based morphometry (VBM) analysis before and after effective 12 weeks sound therapy. The protocol was registered on ClinicalTrials.gov, ID: NCT02774122. In this study, we collected data from 27 matched healthy control (HC) individuals and 27 idiopathic tinnitus patients before and after 12 weeks of sound therapy by using adjusted narrow band sound. 3.0T MRI system and high-resolution 3D structural images were acquired with a 3D-BRAVO pulse sequence. Structural image data preprocessing was performed using the VBM8 toolbox. The Tinnitus Handicap Inventory (THI) score was acquired in the tinnitus group to assess the severity of tinnitus and tinnitus-related distress. Mann–Whitney *U* Test, Wilcoxon Signed-Ranks test, and Pearson’s correlation analysis were used in the statistical analysis. We found significantly decreased gray matter (GM) volume in the left thalami, right thalami, and cochlear nucleus among the tinnitus patients before sound therapy (baseline) compared to the HC group. However, we did not find significant differences in brain regions between the 12-week treatment and HC groups. According to the results of Wilcoxon Signed-Ranks test, the 12-week sound therapy group demonstrated significant greater brain volume compared with the baseline group among these brain regions. Decreased THI score and changed GM volume were not correlated. This is a useful study for observing the characteristics of neuroanatomical changes in patients with idiopathic tinnitus before and after sound treatment. The study characterized the effect of sound therapy on brain volume. It found that sound therapy had a normalizing effect on the bilateral thalami and cochlear nucleus.

**Clinical Trial Registration:** www.ClinicalTrials.gov, NCT02774122.

## Introduction

Tinnitus is a phantom auditory sensation that reduces quality of life for millions worldwide (Shore et al., 2016), affecting approximately 4 to 15% of the population (Baguley et al., 2013). It has been related to listening to loud music (Axelsson and Prasher, 2000), head and neck injuries, sudden sensorineural hearing loss (Pecorari et al., 2020), ototoxic drug use (Dille et al., 2010), and other medical conditions that can affect hearing. Chronic tinnitus can trigger a range of problems, including cognitive problems, sleep disturbances (Schecklmann et al., 2015), depression (Dobie, 2003), and work disorders (Heller, 2003).

According to previous studies (Han et al., 2015; Lv et al., 2016a, b, 2018), tinnitus has been proven to be a symptom characterized by abnormal resting-state functional magnetic resonance imaging (rs-fMRI) in auditory and non-auditory brain regions (Vanneste and De Ridder, 2012) such as the posterior cingulate cortex (Vanneste et al., 2010), insula (van der Loo et al., 2011), parahippocampal region (Vanneste et al., 2011), and anterior cingulate cortex (De Ridder et al., 2011).

Structural changes in the brain have also been explored in tinnitus patients. Voxel-based morphometry (VBM) can quantitatively detect the volume of brain tissue at the voxel level and reflect the differences in the components and characteristics of brain tissue in local brain regions of different groups or individuals (Ashburner and Friston, 2000). Currently, VBM has been used to describe subtle changes in brain structure in tinnitus patients (Husain et al., 2011; Meyer et al., 2016). Studies of patients with right-sided unilateral pulsatile tinnitus have shown that compared with normal controls, patients with unilateral pulsatile tinnitus have a significantly increased gray matter (GM) volume in the bilateral superior temporal gyrus (STG) and significantly decreased volumes in the left cerebellar posterior lobe, left frontal superior orbital lobe (gyrus rectus), right middle occipital gyrus (MOG), and bilateral putamen (Liu et al., 2018).

Effective treatment for tinnitus requires a basic understanding of the functional and anatomical changes in the brain. A recent study in tinnitus patients treated with rTMS demonstrated the reversibility of structural effects after treatment (Poeppl et al., 2018). Another study analyzed the treatment effect on anatomical changes in the brain (Krick et al., 2015). The patients included were in the early stages of disease, with a disease duration of 5.1 weeks. The GM of the precuneus, medial superior frontal areas, and auditory cortex increased in acute tinnitus patients after the Heidelberg model of music therapy intervention, accompanied by significantly decreased tinnitus-related distress. Narrow band noise sound therapy is a commonly used treatment for tinnitus (Henry et al., 2002). Previous studies have demonstrated functional changes in the brain (Han et al., 2019a, b). However, we only found a few related reports on the morphological changes before and after sound therapy with an average treatment time of 1 week (Krick et al., 2015, 2017).

In this study, we applied VBM to analyze the anatomical changes in the brain in patients before and after sound therapy and to explore the morphological feature alterations after treatment. Based on previous studies on structural and functional plasticity, the bilateral thalami are considered to be a critical brain region that is closely associated with effective treatment of tinnitus. In this study, we hypothesize that the brain regions associated with tinnitus, especially the bilateral thalami, may show volume alterations after sound therapy. This study will help to gain deeper insight into the changes in the brain after treatment from a neuroanatomical perspective.

## Materials and Methods

### Standard Protocol Approvals, Registrations, and Patient Consents

This research involved human participants. All authors have declared that this research was approved by the Institutional Review Board (IRB). Written informed consent was obtained from all subjects enrolled in this study. The protocol was registered on ClinicalTrials.gov, ID: NCT02774122.

### Subjects

All patients and healthy volunteers were recruited in our institution. In this study, 27 patients with idiopathic tinnitus were enrolled. The tinnitus sound was described as persistent, high-pitched sound in both of the ears. The inclusion criteria were: (1) 18 to 65 years old; (2) right handedness; (3) tinnitus duration range from 6 to 48 months; (4) no significant hearing loss (hearing thresholds ≥ 25 dB HL at frequencies of 0.250, 0.500, 1, 2, 3, 4, 6, and 8 kHz determined by pure tone audiometry [PTA]); (5) willing to receive sound therapy for 12 weeks and return for reexamination after treatment. The exclusion criteria included: (1) other kinds of tinnitus (such as pulsatile tinnitus), Meniere’s disease, sudden deafness, otosclerosis; (2) neurological signs and/or a history of neurological disease; (3) current chronic medical illness; (4) a history of head trauma; (5) a cardiovascular, pulmonary, or systemic disease; (6) claustrophobia experienced during MRI simulator session. Twenty-seven age-, gender-, education-, and handedness-matched healthy control (HC) subjects were enrolled as normal controls. None of the HCs suffered from tinnitus in the past year. Other exclusion criteria were the same as previously described. The characteristics of the subjects are presented in Table 1.

**TABLE 1:** Demographic and clinical characteristics of participants.

Characteristics	Healthy controls (baseline, *n* = 27)	Tinnitus patients (baseline, *n* = 27)	Tinnitus patients (12th week, *n* = 27)	*P* value
Age (years,*x̄* ± s)	46.6 ± 9.9	46.4 ± 12.0		0.92^*a*^
Years of education	11.8 ± 3.0	11.4 ± 2.5		0.63^*a*^
Gender (male/female)	12/15	12/15		>0.99^*b*^
Center Frequencies	NA	4596.2 ± 1492.9	NA	NA
Tinnitus duration (months)	NA	23.4 ± 10.0		NA
THI score	NA	40.0 ± 9.5	19.0 ± 5.2	<0.001^*c*^
ΔTHI score	NA	21.1 ± 8.3	NA	NA

*Data are presented as mean ± SD for all variables except gender. THI: Tinnitus Handicap Inventory. ΔTHI = THIpre–THIpost. NA: not applicable. ^a^Two-sample t-tests. ^b^Chi-square test. ^c^Paired-samples t-tests.*

### Sound Therapy and Clinical Evaluation

To characterize the tinnitus and prepare for treatment, all of the enrolled tinnitus patients were examined for tinnitus loudness matching (L = loudness of tinnitus), pitch matching (Tf = tinnitus frequency), minimum masking level, and residual inhibition. Narrow band sound therapy was administered to participants in the tinnitus group for 12 weeks, three times a day for 20 min at a time. For each tinnitus patient, the loudness of sound for treatment was set as L + 5 dB. The frequency was set as a 1 kHz narrow band when setting the Tf as the middle point of the delivered sound (Tf ± 0.5 kHz; for example, Tf = 4 kHz, low sound cut = 3.5 kHz, high sound cut = 4.5 kHz). Subjects who could not pitch-match their tinnitus were not included.

We also asked the patients to fill out the Tinnitus Handicap Inventory (THI) to assess the severity of tinnitus before and after treatment. The primary outcome of this prospective study was based on changes in THI score after treatment. A reduction of at least 16 points in the THI was considered effective treatment (Zeman et al., 2011). The HC group was not given any kind of sound during the research.

### Data Acquisition

Images were acquired using a 3.0T GE Signa Excite MR scanner (General Electric Medical Systems, Milwaukee, WI, United States) equipped with an eight-channel, phased-array head coil. All imaging studies were performed at the Medical Imaging Research Center of Beijing Friendship Hospital. Parallel imaging was employed for data acquisition. High-resolution 3D structural images were acquired with a 3D-BRAVO pulse sequence with the following acquisition parameters: TR (repetition time) = 8.8 milliseconds (ms), TE (echo time) = 3.5 ms, TI (inversion time) = 450 ms, field of view (FOV) = 240 × 240 mm, matrix = 256 × 256, and slice thickness = 1 mm without a gap. In total, 196 slices of images were obtained from each subject. We acquired the MRI data of tinnitus patients at baseline and after treatment (week 12). HC individuals only underwent MRI one time.

### Data Preprocessing

Image preprocessing was performed with the VBM8 toolbox in the SPM8 software package (Statistical Parametric Mapping, Wellcome Department of Cognitive Neurology, London, United Kingdom) running in MATLAB (MathWorks, Natick, MA, United States). The procedures for image preprocessing have been described in detail in our previous research (Liu et al., 2018). Briefly, image processing in this work included spatial normalization using the Montreal Neurological Institute’s (MNI) 152 template and segmentation of the GM, white matter (WM), and cerebrospinal fluid (CSF). Only the GM images were analyzed in this study. Modulation was also applied to avoid volumetric deformation of the GM due to stretching and shrinking effects during the normalization procedure. The modulated GM images were smoothed with a 6 mm full width at half maximum (FWHM) isotropic Gaussian kernel. Finally, the smoothed GM images were resampled to a 3 mm × 3 mm × 3 mm voxel size for the statistical analysis. Subjects with excessive head motion (more than 1.5 mm in translation or 1.5° in rotation) were excluded from the analysis.

### Statistical Analysis

Nonparametric test was used in this study. Mann–Whitney *U* Test and Wilcoxon Signed-Ranks test were used to extract GM volumetric changes in this study. A comparison between treated and untreated patients was performed to examine sound therapy-induced effects on structural changes. A *P* value of less than 0.05 was considered statistically significant [false discovery rate (FDR) corrected].

To prove our hypothesis, Pearson’s correlation analyses were further conducted to investigate the relationship between changed GM volume and the clinical characteristics of tinnitus patients (disease duration at baseline, ΔTHI score [ΔTHI score = THIpre–THIpost]). *P* < 0.05 was set as the threshold to determine significance. The GM volume results were visualized with the REST Slice Viewer and BrainNet Viewer^1^ (Xia et al., 2013). Pearson’s correlation analysis was performed using SPSS 17 software (SPSS, Inc., Chicago, IL, United States) between the THI scores.

## Results

### Demographics and Behaviors of Study Participants

In this study, we enrolled 54 subjects, including 27 patients with idiopathic tinnitus. In this group, we applied VBM to analyze anatomical changes in the brain before and after sound therapy. 27 HC individuals were also enrolled. Each group of subjects was age-, sex-, and education-matched (Table 1). THI scores were acquired before and after sound therapy. In the data preprocessing step, none of the subjects were excluded according to the head motion criteria. Significant longitudinal decreases in THI scores were observed. The results are summarized in Table 1.

### Statistical Analysis Results

Significantly decreased GM volume was found in the left and right thalami and cochlear nucleus of the tinnitus patients prior to sound therapy (baseline) compared to participants in the HC group (Table 2).

**TABLE 2:** Regions of significant difference with changed GM volumes in the tinnitus patients compared with the HC group according to the voxel-based morphometry analysis.

Brain Region	Number of voxels	Peak MNI (X, Y, Z)	Nonparametric tests/*P* Value		
			G1 vs. G2	G1 vs.G3	G2 vs. G3
L thalamus	44	−22, −20, 8	0.000^*a*^	0.001^*b*^	0.897^*b*^
R thalamus	31	18, −20, 4	0.000^*a*^	0.001^*b*^	0.622^*b*^
Cochlear nucleus	32	−8, −35, −40	0.012^*a*^	0.001^*b*^	0.095^*b*^

*(P < 0 05, FDR corrected). G1: Group 1 Tinnitus patients (baseline). G2: Group 2 Tinnitus patients (12th week). G3: Group 3 Healthy control. ^a^Wilcoxon Signed-Ranks Test. ^b^Mann–Whitney U Test. Number of voxels: The cluster sizes of the three brain regions, e.g., 44 means this cluster has 44 voxels. MNI: Montreal Neurological Institute. Peak MNI: The specific positioning of each brain region on MIN template.*

**FIGURE 1:**
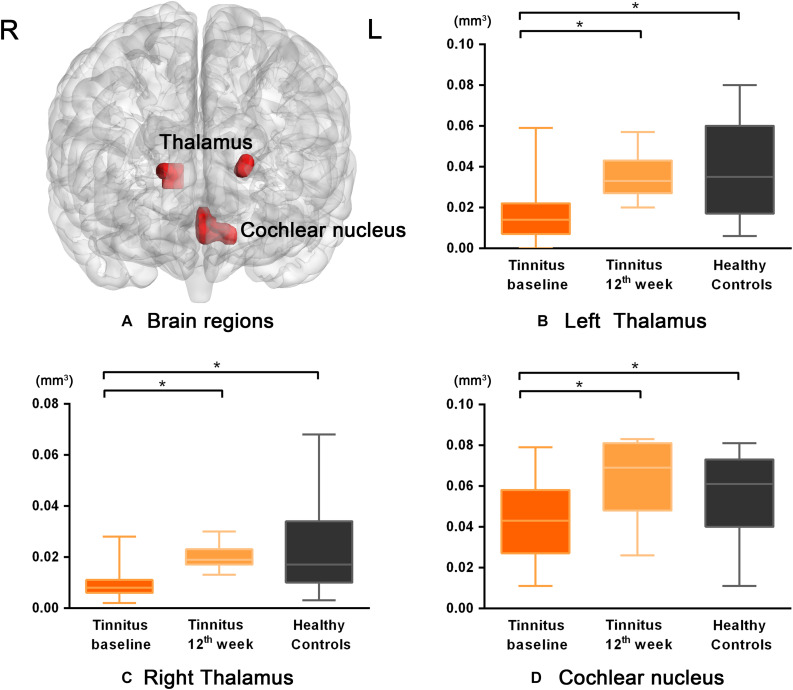
The differences in GM volume among the baseline, after 12 weeks of sound treatment and the healthy control groups (Nonparametric tests; *P* < 0.05, corrected for false discovery rate simulations; L left, R right). **(A)** The bilateral thalamus and cochlear nucleus were the brain regions with statistical differences. Panels **(B–D)** represent the GM volume changes of each brain region in tinnitus baseline after 12 weeks of sound treatment and HC group. Wilcoxon Signed-Ranks test was used to compare tinnitus baseline with after 12 weeks treatment; Mann–Whitney *U* test was used to compare after treatment with normal people, baseline with normal people. *P < 0.05. The results showed differences in GM volume in the left thalamus, right thalamus, and cochlear nucleus, which are shown in red. L, left; R, right; the labeled number corresponds to the number in Table 2. The threshold was set as *P* < 0.05 (corrected). It can be seen that the volume of gray matter increased after 12 weeks treatment compared to baseline and there was no statistically significant difference between the 12 weeks treatment and HC group.

As shown in Figure 1 and Table 2, the statistical analysis demonstrated significant difference in GM volume in the left thalamus, right thalamus, and cochlear nucleus among the tinnitus before sound therapy (baseline), tinnitus after sound therapy (12 weeks), and HC groups.

Compared with the baseline group, the 12-week sound therapy group demonstrated a significantly higher GM volume in all of the regions mentioned above.

Compared with the HC group, the 12-week sound therapy group demonstrated slightly lower GM volume in the bilateral thalami and slightly higher volume in the cochlear nucleus, but these differences did not reach statistical significance.

Compared with the HC group, the baseline group demonstrated a lower GM volume in the bilateral thalami and cochlear nucleus, and there was a significant difference between the two groups.

The results also showed that in the three brain regions described above, the GM volume was higher after 12 weeks of sound therapy than at baseline (Table 3).

**TABLE 3:** VBM-derived brain regions that are critical to VBM prediction, ranked by their changed in treatment time.

Brain Region	Peck MNI (X, Y, Z)	Volume in patients (baseline, *n* = 27)	Volume in patients (12th week, *n* = 27)	Volume in HC (mm^3^)
L thalamus	−22, −20, 8	0.17 (0.08–0.27)	0.39 (0.33–0.51)	0.72 (0.41–0.90)
R thalamus	18, −20, 4	0.10 (0.07–0.13)	0.23 (0.20–0.28)	0.20 (0.12–0.41)
Cochlear nucleus	−8, −35, −40	0.51 (0.32–0.70)	0.83 (0.58–0.97)	0.73 (0.48–0.87)

*Data are presented as quartile for all variables. The GM volume after 12 weeks treatment increased compared with the baseline GM volume, which was closer to the normal subjects.*

### Correlation

No correlation results survived the multiple comparison correction.

## Discussion

This is a meaningful longitudinal investigation that specifically analyzed the changes in GM volume in the bilateral thalami at baseline and after 12 weeks of sound therapy. Anatomical changes in the brain were found in patients before and after sound therapy, mainly in the left thalamus, right thalamus, and cochlear nucleus. Regarding the morphological and functional changes associated with tinnitus, most previous studies and our recent research have focused on functional aspects, including the use of rest-state and task-state fMRI with 7 Tesla MR (Berlot et al., 2020), while research on the structural aspects has been relatively limited, especially for structural changes after treatment. However, there are structural changes in tinnitus patients (Schecklmann et al., 2013) and these changes will affect structure after treatment, so it is necessary to pay more attention to the structural changes associated with tinnitus patients. Some previous studies have mentioned additional functional issues but fewer structural analyses, so the results of this study are important supplements to original research.

In this study, we found that tinnitus patients had structural changes after treatment. Although these changes may not be as significant as functional changes, the results also reflect the efficacy of sound therapy to a certain extent. One reason may be that many previous studies have focused more on functional changes, including local abnormal activities and abnormal activities of the network, than on structural changes (Han et al., 2019b). A recent study using two resting-state functional connectivity (RSFC) approaches to better understand functional network disturbances associated with tinnitus also found that there were many changes in brain function level in tinnitus patients (Leaver et al., 2016). In addition, in recent research performed before and after 12 weeks of sound therapy in tinnitus patients, our team also found that there were more changes in functional connectivity (Lv et al., 2020). A multimodal neuroimaging meta-analysis investigated the neural substrates of tinnitus by combining information from whole-brain VBM studies of GM volume of and ReHo studies of spontaneous brain activity (Cheng et al., 2020). It reported that increased volume of GM in the bilateral STG, right middle temporal gyrus (MTG), and right supramarginal gyrus and decreased GM volume in the bilateral hypothalamus, left superior frontal gyrus (SFG), and right occipital lobe were observed in tinnitus patients (Boyen et al., 2013). Although fewer structural changes have been found in previous studies, structural changes may not be as obvious as functional changes but still have important clinical significance. These brain regions could represent new neuroanatomical features of patients with tinnitus.

The tinnitus treatment options that have been subjected to randomized controlled trials (RCTs) include pharmacological interventions, sound-based interventions, psychological interventions, magnetic stimulation, electrical stimulation, manual physical therapy, relaxation therapy, complementary and alternative medicine (CAM) therapies, and so on (McFerran et al., 2019). In our study, we applied narrow band noise sound therapy. After choosing a suitable treatment method, it is particularly important to pay attention to the duration of the patient’s illness and the time of treatment. These two factors have a great impact on the condition of tinnitus and the relief of the condition. A previous study with the Heidelberg model of music therapy analyzed comparisons of approximately 1 week of treatment and found that there were GM volume changes in the precuneus, medial superior frontal areas, and auditory cortex (Krick et al., 2015). Husain et al. have investigated brain structure in tinnitus patients with a minimally modified version of Mindfulness-Based Cognitive Therapy (MBCT) to treat symptoms of distress associated with tinnitus, which is a cognitive behavior therapy. All participants underwent a complete audiological evaluation during a screening phase and at subsequent pre-intervention (week 0), post-intervention (week 8), and follow-up (week 16) assessments. VBM analysis revealed clusters in bilateral SFG that exhibited significant increases in gray matter volume over the period of intervention and follow-up. Furthermore, gray matter changes in occipital and cingulate regions correlated with declines in tinnitus handicap (Husain et al., 2019). The analysis in our study was performed after 3 months of treatment with narrow band sound therapy, with a longer treatment time and a relatively better treatment effect. Therefore, the morphological changes may be different from those of previous studies, which is supported by our results, although a study reported that the overall evidence for structural abnormalities specifically related to tinnitus is poor at present (Adjamian et al., 2014). Outcomes are divergent and even contradictory across these studies, so we should listen to different opinions, which is conducive to a balanced view of our research.

In this study, it was found that patients with tinnitus had better treatment outcomes, and the brain regions with morphological changes were the bilateral thalami and cochlear nucleus. Our results showed GM volume changes in the bilateral thalami, which also illustrated the importance of the thalamus in tinnitus treatment. Previous research by our team has also found that the thalamus is a very important brain area and it plays a critical role in the perception of tinnitus (Han et al., 2019b). It is also a brain relay area that regulates sensory information flow to and from the auditory cortex (Rauschecker et al., 2010; Leaver et al., 2011). A study reported that thalamic regional expansion may signify dysfunctional auditory gating in the thalamus, where inhibition of tinnitus signals at the level of the thalamus is disrupted due to abnormal changes in the limbic system, ultimately leading to the perception of tinnitus (Tae et al., 2018). This is consistent with the results of this study. In addition to the thalamus, there are also some other important brain regions that affect tinnitus. Although they did not show morphological changes after treatment, they have functional changes and have been found in our previous research. Therefore, the results also show that structure and function do not necessarily change simultaneously, and changes between the two should be analyzed objectively.

The cochlear nucleus is referred to as a single entity but is the first relay station of the brainstem to receive auditory pulses. The cochlear nucleus is divided into the ventral cochlear nucleus (VCN) and the dorsal cochlear nucleus (DCN). The cochlear nerve further divides the ventral nucleus of the cochlea into an anteroventral cochlear nucleus (AVCN) and posteroventral cochlear nucleus (PVCN) (Mishra et al., 2018). These direct synaptic connections from one cochlear nucleus to the other could play a significant role in non-lateralized tinnitus (Levine and Oron, 2015). Our results are mostly concentrated on the left DCN. We will continue to study the role of the cochlear nucleus in tinnitus in the next experiments.

### Limitations

There are several limitations in this study. First, the current sample size is small. There was no relationship between changes in GM volume and THI scores in tinnitus patients, which may be due to the small sample size in this study. Further studies should include a larger sample size, such as more than a hundred patients, which would improve the results. Of course, collecting so many subjects for treatment is also a challenge. Second, this study did not include a sham treatment group, so there may be some placebo factors that cannot be completely ruled out. Third, we only used the baseline data of HC group as control group. In the next study, we will also follow up the HCs with the same time point. Fourth, the tinnitus patients included in this study did not have significant hearing loss, which represents only a portion of patients with different types of tinnitus. Fifth, subjects who could not pitch-match their tinnitus were not included. Lastly, although many studies have used VBM to better understand tinnitus, the results may be inconsistent. There is a pressing need to standardize the use of VBM when evaluating tinnitus patients (Scott-Wittenborn et al., 2017). We should pay further attention to tinnitus heterogeneity, which could be expressed in terms of treatment response (Simoes et al., 2019). Research using machine learning may be helpful.

## Conclusion

This study analyzed the anatomical changes in tinnitus patients before and after treatment for 3 months. The effects of sound therapy includes alterations in brain volume, especially in the bilateral thalami.

## Data Availability Statement

The raw data supporting the conclusions of this article will be made available by the authors, without undue reservation.

## Ethics Statement

This experiment was approved by the Institutional Review Board (IRB) of Beijing Friendship Hospital, Capital Medical University, Beijing, China. Written informed consent was obtained from all subjects enrolled in this study. The protocol was registered on ClinicalTrials.gov, ID: NCT02774122. The patients/participants provided their written informed consent to participate in this study. Written informed consent was obtained from the individual(s) for the publication of any potentially identifiable images or data included in this article.

## Author Contributions

XW designed the experiments, performed the statistical analysis, and wrote the manuscript. PR and YL conducted the statistical analysis. PZ and PFZ contributed to the manuscript revision. HL, ZW, CL, and QC also collected the data. SG, ZY, and ZCW are guarantors of this work. HL and ZCW are the corresponding authors of this manuscript. They have full access to all the data in the study and take responsibility for the integrity of the data and the accuracy of the data analysis. All authors contributed to the article and approved the submitted version.

## Conflict of Interest

The authors declare that the research was conducted in the absence of any commercial or financial relationships that could be construed as a potential conflict of interest.

## Footnotes

1 http://www.nitrc.org/projects/bnv/
